# An unusual location of solitary fibrous tumor in heart—A case report and review of literature

**DOI:** 10.1002/cnr2.1698

**Published:** 2022-08-30

**Authors:** Kristen Fain, Kanak Parmar, Meenu Sharma, Robert Horn, Pooja Sethi

**Affiliations:** ^1^ School of Medicine Texas Tech University Health Science Center Lubbock Texas USA; ^2^ Department of Internal Medicine Texas Tech University Health Science Center Lubbock Texas USA; ^3^ Department of Pathology Texas Tech University Health Science Center Lubbock Texas USA; ^4^ Department of Hematology‐Oncology Texas Tech University Health Science Center Lubbock Texas USA; ^5^ Department of Cardiovascular Medicine Texas Tech University Health Science Center Lubbock Texas USA

**Keywords:** cardiac tumor, pericardium, solitary fibrous tumor

## Abstract

**Background:**

Solitary fibrous tumor (SFT) are rare spindle cell tumors originating from the mesenchymal cells mostly from the visceral pleura. SFT was first described as a distinct entity in 1931 by Klemperer et al. Until now, we have limited data regarding the manifestation and behavior of extra pleural forms such as cardiac SFT. Here we present a case of SFT involving the pericardium where the diagnosis was made by imaging followed by biopsy findings. We also review the literature of SFT involving the heart and the management approaches.

**Case Presentation:**

An 81‐year‐old male presented with progressive dyspnea. Computed tomography (CT) of the chest showed a 6.2 × 5.3 cm soft tissue mass in the anterior mediastinum. Further imaging with CT angiogram showed a stalk‐like connection to the pericardium. A biopsy of the mass showed spindle cells positive for BCL‐2, CD34, and STAT 6, indicative of a solitary fibrous tumor. A surveillance approach was adopted for the patient.

**Conclusion:**

Primary pericardial tumors are exceedingly rare, with a prevalence rate of 0.001%‐0.007%. Diagnosing a SFT requires a positive CD34 and BCL‐2 marker. The current recommendation is resection of localized disease which has been documented to be curative in cases of benign disease however our patient was put on surveillance.

## 1 INTRODUCTION

Solitary fibrous tumor (SFT) is a rare spindle cell tumor derived from mesenchyme, first described in 1931 by Klemperer et al. originating from the pleura.^1^ In 1942, Stout et al., described hemangiopericytomas which was thought to be a vascular neoplasm related to smooth muscle perivascular cells known as pericytes.^2^, ^3^ Given the phenotypic and behavioral overlap hemangiopericytoma and SFT were subsequent described to be one tumor type by pathologist. They mostly originate from the pleura however, extra‐pleural locations including deep soft tissue, peritoneum, mediastinum, bones, orbit, and parotid glands have been reported. Cardiac SFT are exceedingly rare with limited cases of SFT involving the pericardium documented in the literature.^4^, ^5^, ^6^, ^7^, ^8^ Chest radiography, echocardiography, computed tomographic scan (CT scan), positron emission tomography (PET)‐CT and magnetic resonance imaging (MRI) are each useful in the diagnosis and origin of the cardiac SFTs.^9^ Pericardial and epicardial cases of cardiac SFTs are mostly benign with only four reported malignant cases.^7^, ^9^, ^10^, ^11^ The best treatment for SFT is surgical resection with a goal of tumor negative surgical margins; however, there is some role for radiotherapy in recurrent SFTs. Chemotherapy is reserved either for metastatic disease or for symptomatic nonresectable SFTs.^7^ Here we present a case of SFT involving the heart where the diagnosis was made by imaging followed by biopsy findings. We also review the literature of SFT involving the heart and the management approaches.

## 2 CASE PRESENTATION

**FIGURE 1:**
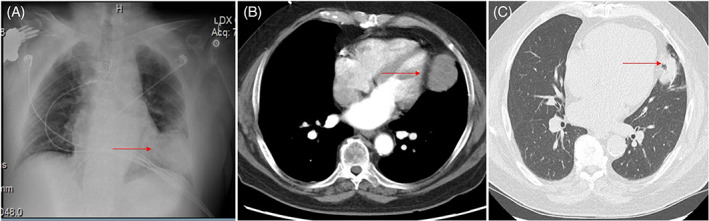
(A) Chest X‐ray showing a rounded mass measuring approximately 8 cm in left lower lobe. (B) CT chest showed a 6.6 cm enhancing soft tissue mass within the left lower mediastinum. A focal point measuring approximately 1 cm can be seen where there does not appear to be a fat plane of separation from the left ventricular myocardium. (C) CT heart angiogram showed a 6.9 by 5.4 cm mass in left inferior mediastinum with a stalk‐like connection to pericardium.

**FIGURE 2:**
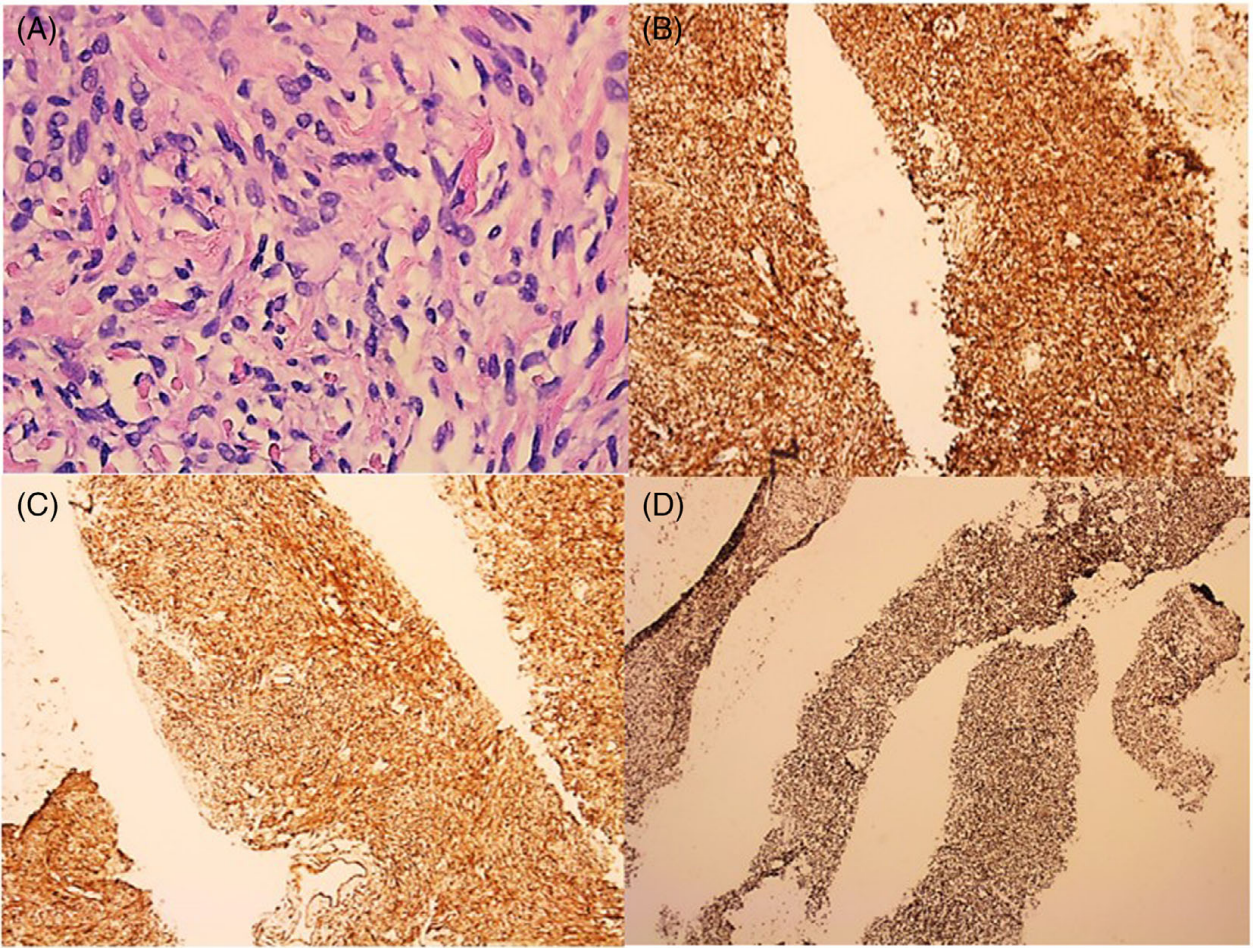
(A) Microscopy of tissue specimen stained with hematoxylin and eosin stain showing spindle cells. (B) Immunohistochemical stains at showing tumor to be BCL‐2 positive. (C) Immunohistochemical stains showing tumor to be CD34 positive. (D) Immunohistochemical stains showing tumor to be STAT 6 positive. (A. 40× original magnification, B. 5× original magnification, C. 5× original magnification and D. 2.5× original magnification)

A 81‐year‐old male presented with 4 months of progressively worsening dyspnea on exertion. Medical history was significant for chronic kidney disease (CKD), coronary artery disease status post coronary artery bypass graft (CABG). On presentation, blood pressure was 138/82 mm Hg, heart rate was 76 beats per minute, and a normal respiratory rate. Electrocardiogram (EKG) was normal. Chest radiography (CXR) revealed a rounded mass measuring 8 cm in the left lower lung lobe (Figure 1A). CT scan of the chest without contrast showed an enhancing soft tissue mass along the left aspect of the pericardium, measuring 6.2 × 5.3 cm (Figure 1B). At this time, differential diagnosis included pericardial cyst, teratoma, lymphangioma, branchial cyst. CT heart angiogram was ordered and revealed a 6.9 × 5.4 cm mass in the left inferior mediastinum with a stalk like connection to the pericardium (Figure 1C). A biopsy of the mass was performed. Pathology revealed spindle cells (Figure 2A). Immunohistochemical (IHC) stains revealed a BCL‐2, CD34, and STAT 6 positive, indicative of a solitary fibrous tumor (Figure 2B, C, D). IHC stains S100, CD3, CD5, CD 20, CD 117, CK34 Beta E12, SOX 10, p40, desmin, terminal deoxynucleotidyl transferase (TDT), smooth muscle actin, and pan cytokeratin were negative. Ki‐67 was 3% (low). Cardiothoracic surgery was consulted. After shared decision making with the patient a surveillance approach was adopted. On follow up 6 months later, patient had developed atrial fibrillation however the tumor size has remained stable.

## 3 DISCUSSION

SFTs have been reported in the literature since 1931 and have been previously classified as hemangiopericytomas.^1^ In the beginning, SFT was thought to be of mesothelial origin; however, reports of extra pleural SFT originating from serous membranes suggested that SFT could arise from submesothelial mesenchymal elements. We report a case of SFT arising from the pericardial membrane. Due to the rare nature of these infrequently metastasizing fibroblastic, spindle cell mesenchymal tumors, the literature about them mainly consists of case reports and retrospective reviews.

SFTs most commonly originate in the pleura, with 80% originating from the visceral pleura and 20% arising from the parietal pleura. However, extra pleural locations such as the abdomen, soft tissue, mediastinum, pelvis, meninges, extra cranial tissue, and retroperitoneum have been documented.^7^ Cardiac localization of this tumor is exceedingly rare. In a literature review by Zhang et al., 3 cases were malignant, 10 cases were benign, and 2 cases were unknown.

Our PubMed search from inception till January 2022, showed most cases reported to be of pericardial origin of cardiac SFT and few reported from the epicardium as shown in Table 1.^2^, ^5^, ^6^, ^7^, ^8^, ^9^, ^10^, ^12^, ^13^, ^14^, ^15^, ^16^, ^17^, ^18^, ^19^, ^20^, ^21^, ^22^, ^23^, ^24^, ^25^, ^26^, ^27^ The most accurate predictors of malignancy in SFT on histopathology is nuclear pleomorphism, cellular atypia, necrosis, high cellularity, hemorrhage, cystic degeneration, crowding and overlapping of nuclei within the cytoplasm, a diameter 10 cm or more than 4 mitoses per 10 high‐power fields.^4^, ^9^, ^28^ Our case was considered benign based on the lack of mitotic activity and no necrosis. Recent speculation surrounding markers p53 and Ki67 and their ability to predict the malignant potential of SFTs has gained attraction; however, this is not universally accepted at this time.^28^ Though these markers are controversial, our patient had a low Ki67 of 3% on the pathology report, which strengthened the prediction for lower malignant potential in our case.

**TABLE 1:** Reported cases of cardiac solitary fibrous tumors in the literature till January 2022

Number	Author, Year	Age(years)	Gender	Chief Complaint	Location	Malignancy	Treatment	Prognosis
1	Roggli, 1987^14^	NA	NA	NA	Pericardium	Benign	N/A	N/A
2	El‐Naggar et al., 1989^6^	56	F	Incidental	Pericardium	Benign	Surgical resection	Recurrence, alive
3	Bortolotti et al., 1992^12^	60	M	Fatigue, anemia, chest discomfort, dyspnea	AA, PT	Benign	Surgical resection	Alive, well
4	Segawa et al., 1995^15^	50	F	Dyspnea, palpitations	RV	NA	Surgical resection	Alive well
5	Burke et al., 1996^16^	NA	NA	NA	Pericardium	Benign	NA	N/A
6	Flemming et al., 1996^17^	53	F	Could not interpret due to language barrier	LV	NA	NA	No meatastasis, died post tx
7	Andreani et al., 1998^18^	60	M	Exertional dyspnea	Pericardium	Benign	Surgical resection	No recurrence, alive
8	Odim, 2003^20^	NA	NA	NA	NA	Benign	NA	No recurrence
9	Corgnati G, 2004^19^	30	M	NA	AA, PT	Benign	NA	No recurrence, alive
10	Bothe W, 2005^21^	39	F	Palpitations	RA	Benign	Surgical resection	Alive, well
11	Croti UA, 2008^22^	5 months	M	Infant RDS	LA	Benign	Surgical resection	Died, non‐cardiac
12	Zhao XG, 2012^9^	55	M	Chest distress and dyspnea	RA	Malignant	Surgical resection	Operative Death
13	Taguchi S, 2013^10^	49	F	N/A	LV	Malignant	Surgical resection	Alive, well
14	Bianchi G, 2013^2^	68	F	Dyspnea, fatigue	LV	Benign	Surgical resection	Alive, well
15	Tamenishi A, 2013^23^	30	F	Syncope	LPA	Benign	Surgical resection	Alive, well
16	Ge D, 2015^26^	NA	NA	NA	NA	NA	NA	NA
17	Xiang, 2017^11^	42	M	Dry cough	Pericardium	Malignant	Surgical resection	Recurrence, died w/ no tx
18	Shao et al. 2019^5^	51	F	Symptoms of heart failure	Pericardium	Benign	NA	NA
19	Zhang et al. 2019^7^	64	F	Exertional dyspnea and abdominal distention	RA pericardium	Malignant	Pericardial drainage and surveillance	NA
20	Sheikhey et al. 2021^8^	44	F	Exertional dyspnea and generalized edema	Intrapericardial	Benign	Surgical resection	Alive, well
21	Suzuki et al. 2021^27^	24	F	Incidental finding	LA endocardium	Malignant	Surgical resection	Metastatic, died post tx
22	Our case	81	M	Exertional dyspnea	Pericardium	Benign	Surveillance	No recurrence, alive

Abbreviations: 5 M, five months; AA, ascending aorta; F, female; LA, left atrium; ltPA, left pulmonary artery; LV, left ventricle; M, male; NA, not available; PT, pulmonary trunk; RA, right atrium; RDS, respiratory distress syndrome; RV, right ventricle; Tx, heart transplantation; tx, treatment.

SFTs are most commonly present in adults aged 50–60 years old. To date, there is no known association with exposure to asbestos, tobacco, radiation, or other toxins. Our patient had coronary artery disease and underwent CABG, however there is no literature reporting any association between this comorbidity and SFT. Symptoms at presentation are largely dependent on the location of the tumor. Patients with thoracic SFTs commonly experience dyspnea, cough, syncope, peripheral edema, chest pain, and palpitations. However, 64% of cases were reported to be asymptomatic. Pericardial SFTs present most commonly with exertional dyspnea.^4^

Advances in immunohistochemistry (IHC) and cytogenetics have allowed pathologists to more accurately diagnose SFTs. IHC markers such as CD34, Bcl2, CD99, vimentin, and STAT 6 are often expressed in SFTs. Studies reveal that the diagnosis of SFT requires a positive CD34 and Bcl2 marker.^28^ 95%–100% of all SFT cases presented with a positive CD34 marker on IHC analysis.^1^ However, STAT 6 has been noted to be highly sensitive and specific, at 98% sensitivity and 85% specificity for SFTs by Schweizer et al. in meningeal hemangiopericytoma.^29^ In our case, there was initial suspicion for a pericardial cyst following imaging findings of a homogenous, thin‐walled mass. However, biopsy revealed spindle cells and immunohistochemical markers positive for CD34, STAT 6, and Bcl2 confirmed the diagnosis of SFT.

To our knowledge, guidelines for the management of thoracic SFTs have not been established. The current recommendation is resection of localized disease which has been documented to be curative in cases of benign disease.^4^ Of note, it is important to emphasize that surgical resection of cardiac SFTs can be complicated by close proximity to vascular structures. Complete resection should be prioritized over preservation of vascular and nerve structures in order to prevent recurrence.^15^ Though malignant thoracic SFTs are strongly associated with recurrence, benign SFTs can be aggressive in nature and necessitate complete resection if when possible.^5^ FDG‐PET of whole body was demonstrated by Shao et al. to rule out metastasis.^5^ In our case given prior history of CABG, tumor location, and patient preference a decision was made to monitor his tumor.

In conclusion, primary pericardial tumors are exceedingly rare, with a prevalence rate of 0.001%–0.007%.^2^, ^12^ Diagnosing a SFT requires a multifaceted approach; with IHC and histology being essential for diagnosis. Our case highlights the importance of considering rare diagnosis when working up a mediastinal mass.

## INFORMED CONSENT

Written consent from the patient was obtained for the publication of case details and use of images.

## AUTHOR CONTRIBUTIONS

**Kristen Fain:** Conceptualization (equal); data curation (equal); writing – original draft (equal). **Kanak Parmar:** Conceptualization (equal); writing – original draft (equal); writing – review and editing (lead). **Meenu Sharma:** Resources (equal). **Pooja Sethi:** Writing – review and editing (equal).

## FUNDING INFORMATION

No funds were required for the study.

## CONFLICT OF INTEREST

The authors state there are no conflicts of interest regarding the publication of this article.

## Data Availability

Data sharing is not applicable to this article as no new data were created or analyzed in this study.
